# A Death Effector Domain Chain DISC Model Reveals a Crucial Role for Caspase-8 Chain Assembly in Mediating Apoptotic Cell Death

**DOI:** 10.1016/j.molcel.2012.05.004

**Published:** 2012-07-27

**Authors:** Laura S. Dickens, Robert S. Boyd, Rebekah Jukes-Jones, Michelle A. Hughes, Gemma L. Robinson, Louise Fairall, John W.R. Schwabe, Kelvin Cain, Marion MacFarlane

**Affiliations:** 1 MRC Toxicology Unit, Hodgkin Building, P.O. Box 138, Lancaster Road, Leicester LE1 9HN, UK; 2 Henry Wellcome Laboratories of Structural Biology, Department of Biochemistry, University of Leicester, Lancaster Road, Leicester LE1 9HN, UK

## Abstract

Formation of the death-inducing signaling complex (DISC) is a critical step in death receptor-mediated apoptosis, yet the mechanisms underlying assembly of this key multiprotein complex remain unclear. Using quantitative mass spectrometry, we have delineated the stoichiometry of the native TRAIL DISC. While current models suggest that core DISC components are present at a ratio of 1:1, our data indicate that FADD is substoichiometric relative to TRAIL-Rs or DED-only proteins; strikingly, there is up to 9-fold more caspase-8 than FADD in the DISC. Using structural modeling, we propose an alternative DISC model in which procaspase-8 molecules interact sequentially, via their DED domains, to form a caspase-activating chain. Mutating key interacting residues in procaspase-8 DED2 abrogates DED chain formation in cells and disrupts TRAIL/CD95 DISC-mediated procaspase-8 activation in a functional DISC reconstitution model. This provides direct experimental evidence for a DISC model in which DED chain assembly drives caspase-8 dimerization/activation, thereby triggering cell death.

## Graphical abstract

**Graphical Abstract:**

**Figure:**
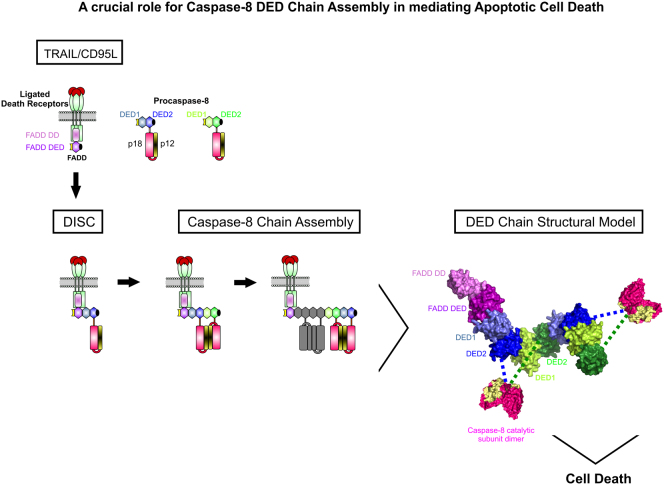

**Highlights:**

► The TRAIL DISC is a soluble >700 kDa complex of TRAIL-Rs, FADD, and DED-only proteins ► LC-MS/MS defines FADD as substoichiometric relative to TRAIL-Rs/DED-only proteins ► Structural modeling reveals that FADD recruits multiple DED-only proteins to the DISC ► We uncover a crucial role for caspase-8 DED chain assembly in triggering cell death

## Introduction

Apoptosis is a highly regulated and morphologically distinct form of cell suicide that enables multicellular organisms to remove unneeded, damaged, or infected cells. Apoptosis plays important roles in embryonic development and adult tissue maintenance, and its deregulation contributes to cancer, as well as neurodegenerative and autoimmune diseases. Two distinct signaling pathways induce apoptosis: the intrinsic pathway, activated by intracellular stress/damage, and the extrinsic pathway, initiated extracellularly by ligation of “death receptors” which are a subset of the tumor necrosis factor (TNF) receptor superfamily. TNF-related apoptosis-inducing ligand (TRAIL), unlike other TNF family ligands such as CD95L, can selectively induce apoptosis in tumor cells, making it an attractive candidate for targeted cancer therapy. TRAIL initiates the extrinsic pathway of apoptosis by ligating the signaling competent TRAIL receptors, TRAIL-R1/TRAIL-R2, triggering formation of a multiprotein death-inducing signaling complex (DISC) which is also formed after CD95 ligation. The current model for DISC formation proposes that ligation of CD95 or TRAIL-R1/TRAIL-R2 triggers recruitment of the adaptor molecule FADD via a homotypic interaction between the death domains (DDs) within the receptor and FADD. The death effector domain (DED) of FADD then recruits DED-only proteins (procaspase-8/procaspase-10 or c-FLIP), forming an active DISC. The DISC enables activation of the initiator procaspase-8/procaspase-10, a process that requires both proximity-induced dimerization and proteolytic cleavage (Hughes et al., 2009; Oberst et al., 2010). Once activated, caspase-8/caspase-10 initiate the caspase cascade, directly through cleavage of procaspase-3 or indirectly via Bid cleavage, ultimately resulting in apoptosis. Intriguingly, in the presence of caspase inhibitors or following caspase-8 gene ablation, death receptors have also recently been shown to induce necrotic cell death, a process which is dependent on the kinase activity of RIPK1 and RIPK3 (Cho et al., 2009; He et al., 2009; Zhang et al., 2009).

Originally, in the DISC, it was proposed that one ligand trimer binds to one receptor trimer, resulting in recruitment of three molecules of FADD and three molecules of procaspase-8. A structure-based model of a trimeric CD95-FADD interaction has been reported (Weber and Vincenz, 2001), but this does not accommodate the requirement for dimerization in initiator caspase activation (Boatright et al., 2003; Donepudi et al., 2003). This, and a difficulty in demonstrating a 1:1 interaction between CD95 DD and FADD DD in vitro, led to the suggestion of a 3:2:2 ratio for the CD95:FADD:caspase-8 complex (Berglund et al., 2000). However, the discovery that hexameric, and not trimeric, CD95 ligand is required for DISC formation (Holler et al., 2003) indicated that the DISC is not formed as individual trimers but instead forms clusters. Current theories propose that three receptors recruit three FADD molecules, which in turn recruit three initiator caspase or c-FLIP molecules; these trimeric complexes are then stitched together by CD95 (Scott et al., 2009) and/or FADD interaction (Carrington et al., 2006) to produce a “honeycomb” structure. Since the bridges between trimeric complexes contain two molecules of FADD, this would provide two initiator caspase binding sites, possibly explaining how dimerization of caspase-8 could be achieved by trimeric receptors. However, this model of DISC formation may be too simplistic, since recent structural work on the interaction of the DDs of CD95 and FADD have indicated that the stoichiometry between these domains is predominantly 5:5 (Esposito et al., 2010; Wang et al., 2010). Importantly, neither the stoichiometry of the core components nor the structure of the native DISC has ever been fully defined with full-length proteins. Thus, there is considerable confusion concerning the proteomic composition, localization, and structure of the native DISC.

In this study we have reinvestigated the mechanisms that regulate assembly of this key multiprotein signaling complex. Using sucrose gradient centrifugation, we now show that the majority of TRAIL DISC formed in a panel of hematopoietic cell lines is a >700 kDa complex not associated with lipid rafts (LRs). Furthermore, using label-free quantitative mass spectrometry we have probed the stoichiometry of the proteins that constitute the DISC and show that the native DISC does not conform to the generally accepted 1:1:1 model of receptor:FADD:caspase-8/caspase-10 or c-FLIP; instead, the adaptor protein FADD is consistently substoichiometric compared to either TRAIL-Rs or caspase-8. Based on these striking observations, we propose an alternative DISC structural model which relies on caspase-8 DED chain formation. Importantly, we provide direct experimental evidence to show caspase-8 DED chain formation within the complex and demonstrate that preventing caspase-8 chain formation blocks activation of the caspase cascade and cell death. In view of the emerging role of caspase-8 in determining prosurvival or prodeath signals within a number of signaling complexes that assemble upon activation of cell surface receptors (e.g., TNFR1, CD95, TRAIL-R, TLR3, TLR4), as well as intracellular sensors/adaptors (RIG-1) (Bertrand and Vandenabeele, 2011; Festjens et al., 2007), this paradigm-changing model for DISC formation has far-reaching implications in terms of our understanding of how key multiprotein complexes regulate cytokine signaling outcome in both normal cell physiology and disease.

## Results

### TRAIL DISC Is Predominantly Comprised of TRAIL-Rs, FADD, and DED-Only Proteins

**Figure 1:**
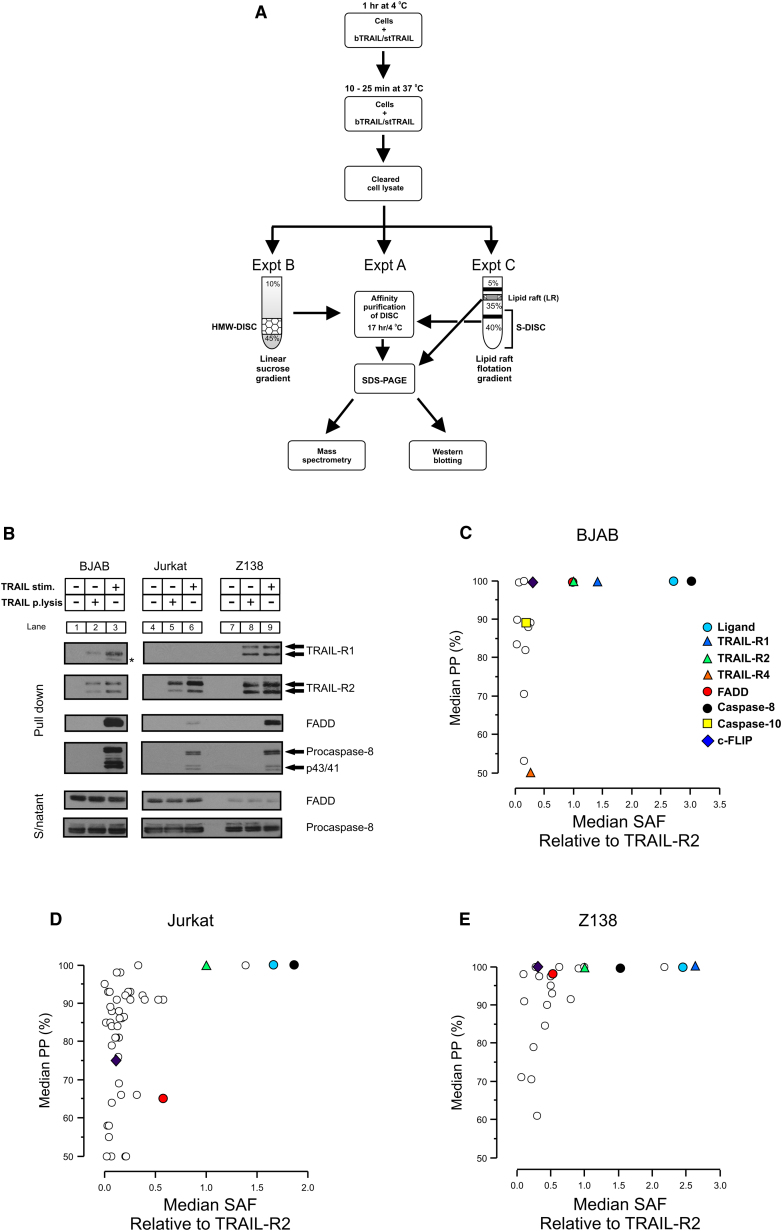
Western Blot and LC-MS/MS Analysis of TRAIL DISC Composition in BJAB, Jurkat, or Z138 Cells (A) Schematic for activation and affinity purification of total, HMW, or soluble TRAIL DISC: BJAB, Jurkat, or Z138 cells were stimulated with either biotin-labeled (bTRAIL) or Strep-II-tagged (stTRAIL) TRAIL under conditions that induced maximal DISC formation. Cleared cell lysates (Figure S1) were subjected to one of three protocols (Expt A, Expt B, or Expt C). Expt A allowed the purification of all ligand-bound complexes, and Expt B was used to separate low molecular weight ligand-receptor complexes from high molecular weight (HMW) DISC, while Expt C permitted independent isolation and analysis of lipid raft-associated (LR) fractions and soluble TRAIL DISC (S-DISC). Bead-captured complexes and LR fractions were separated by SDS-PAGE and analyzed by mass spectrometry or western blotting. (B) Cells were stimulated with bTRAIL for 1 hr at 4°C and further incubated at 37°C for 10 (BJAB) or 25 (Jurkat and Z138) min, lysed, and the TRAIL DISC isolated. DISC-containing eluates and cleared lysate supernatants (1% of total input) were separated by SDS-PAGE for western blot of the known components. Film exposures for each protein (DISC and supernatants) are the same across the three cell lines except in the case of FADD, where 10-fold longer film exposures were required for detection of FADD in the DISC compared to supernatants. Asterisk denotes residual signal from caspase-8 antibody. TRAIL stim., TRAIL treated; TRAIL p.lysis, TRAIL added to cleared lysates to capture TRAIL-Rs. (C–E) Graphical analysis of TRAIL DISC mass spectrometry data. Proteins identified by shotgun proteomics of TRAIL DISC purified from BJAB (C), Jurkat (D), or Z138 (E) cells that fulfilled the necessary criteria were plotted according to median protein probability (PP) and median spectral abundance factor (SAF) relative to TRAIL-R2. n = 3 for BJAB and Jurkat, n = 6 for Z138.

In this study we characterized the native TRAIL DISC formed in a panel of hematopoietic tumor cell lines derived from either B or T cell leukemias. Jurkat, BJAB, and Z138 cells were treated with biotin-labeled/Strep-tagged TRAIL and the resulting DISC affinity purified (Figure 1A, Expt A) and analyzed by western blotting (Figure 1B) or mass spectrometry (Figures 1C–1E). All three cell lines formed variable amounts of TRAIL DISC, comprising TRAIL-R1/TRAIL-R2, FADD, and procaspase-8 (Figure 1B). Jurkat cells contained approximately ten times less TRAIL DISC than BJAB cells (Figure 1B, lanes 3 and 6) and did not contain TRAIL-R1, consistent with previous reports that TRAIL-R1 is not present on the surface of Jurkat cells (MacFarlane et al., 2002; Sprick et al., 2000).

We then analyzed the purified complexes and unstimulated receptor controls by LC-MS/MS, and more than 1,000 proteins were identified. Further bioinformatic analysis (see the Experimental Procedures) produced a much smaller list of potential DISC components and interactors. All known TRAIL DISC components (TRAIL-R1/TRAIL-R2, FADD, caspase-8/caspase-10, c-FLIP) were identified with high confidence, multiple peptide matches, and good coverage of the protein sequences (Figure S2A). Furthermore, plotting the spectral abundance factor (SAF) of each identified protein (relative to TRAIL-R2) against the protein probability illustrated that the known components were identified with the highest statistical confidence in all three cell types (Figures 1C–1E). TRAIL-R1 was identified in the TRAIL DISC from BJAB (Figure 1C) and Z138 (Figure 1E) cells but not in Jurkat cells (Figure 1D), in agreement with western blotting (Figure 1B) and cell surface expression data (MacFarlane et al., 2002). The highest amount of TRAIL DISC was detected in BJAB cells (Figure 1B), and additional components, namely TRAIL-R4 and caspase-10, were also identified by LC-MS/MS in these cells (Figure 1C).

**Figure 2:**
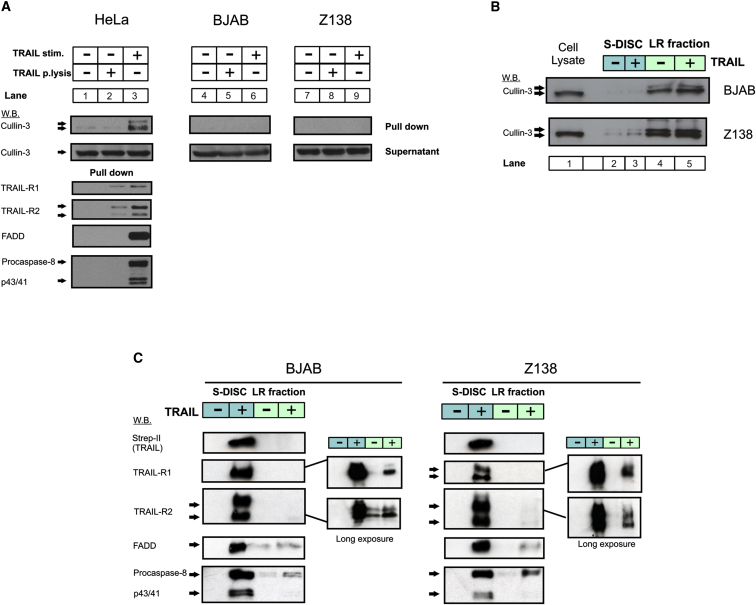
TRAIL DISC Is Predominantly Formed in the Soluble Cellular Fraction Rather Than in Lipid Rafts (A) Cullin-3 is associated with TRAIL DISC formed in the epithelial HeLa cell line. HeLa, BJAB, and Z138 cells were stimulated with bTRAIL for 1 hr at 4°C and further incubated at 37°C for 10 (BJAB) or 25 (Z138 and HeLa) min and the TRAIL DISC isolated. DISC-containing eluates were separated by SDS-PAGE for western blot. Blots for the known components of the BJAB and Z138 DISC are shown in Figure 1B. Exposure times for cullin-3 are the same across the three cell lines. (B) Cullin-3 is predominantly found in the LR fraction (green) of hematopoietic cell lines and is not associated with S-DISC (blue). Z138 or BJAB cells were stimulated with bTRAIL (1 hr at 4°C and 25 min at 37°C) and the lysate separated into soluble and lipid raft fractions. Equivalent quantities of the LR fraction or TRAIL DISC isolated from the soluble fraction (S-DISC) were subjected to SDS-PAGE and western blotting for cullin-3. (C) TRAIL DISC is predominantly found in the soluble fraction of hematopoietic cell lines. Samples prepared as in (B) were analyzed for core DISC components by western blotting. Blots shown are representative of n = 3.

Intriguingly, despite reports of additional proteins including RIP1 being associated with either the CD95 or TRAIL DISC (Harper et al., 2001; reviewed in Peter and Krammer, 2003), in the hematopoetic tumor cell lines tested here, only the core components, TRAIL-Rs, FADD, and DED-only proteins (caspase-8/caspase-10 and c-FLIP), were consistently identified as bona fide DISC components. Additional proteins were identified, although generally at a lower level of confidence than the known DISC components; these may represent new DISC interactors and are currently under investigation. More recently, cullin-3 has been reported to be a TRAIL DISC interactor in H460 (lung tumor) cells, where it is involved in the polyubiquitination of caspase-8, resulting in p62-mediated aggregation (Jin et al., 2009). Cullin-3 was not detected by mass spectrometry (Figure 1) or immunoblotting (Figure 2A, lanes 6 and 9; data not shown) in the TRAIL DISC purified from Z138, BJAB, or Jurkat cells. However, cullin-3 was detected in the TRAIL DISC isolated from HeLa cells (Figure 2A, lane 3). Since BJAB and Z138 cells contained significant amounts of cullin-3, we investigated the subcellular distribution of cullin-3. Strikingly, western blot analysis of LR and soluble DISC (S-DISC) fractions (Figure 1A, Expt C) showed that cullin-3 predominantly localized to the LR fraction in both unstimulated and TRAIL-treated cells (Figure 2B, lanes 4 and 5), whereas core DISC components (TRAIL-R1/TRAIL-R2, FADD, and caspase-8) were only detected in the S-DISC fraction (Figure 2C). Importantly, the LR proteins Flotillin-1 and Lyn were present in the LR fraction but absent in the S-DISC (Figure S3), and, significantly, were not detected by LC-MS/MS analysis of the DISC. Even with high film exposures, only very small amounts (<5% of total) of TRAIL-R1/2 could be detected in the LR fraction (Figure 2C, panel inset), potentially explaining the absence of an interaction between LR-associated cullin-3 and the hematopoetic DISC. Thus, the role of cullin-3 and LRs in TRAIL DISC formation/function is both context and cell-type dependent and may be more relevant in epithelial-derived tumors than those of hematological origin.

### Mass Spectrometry of the TRAIL DISC Reveals that FADD Is Substoichiometric

**Figure 3:**
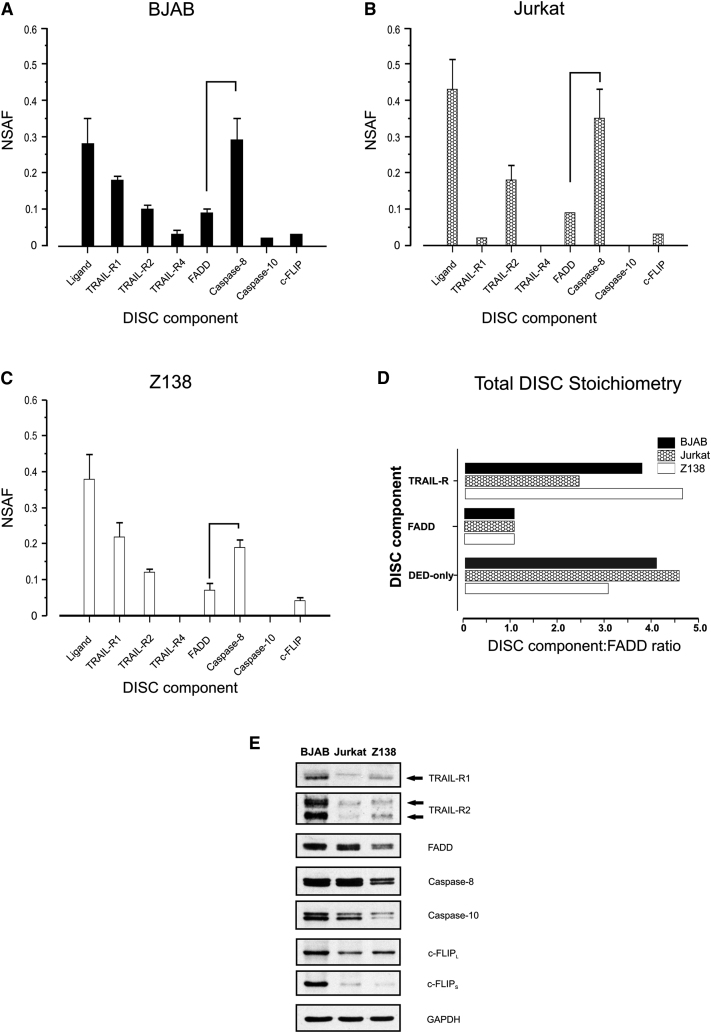
Mass Spectrometry Reveals an Alternative Stoichiometry for the TRAIL DISC (A–C) Normalized spectral abundance factor (NSAF) analysis of mass spectrometry data obtained for the TRAIL DISC isolated from total BJAB (A), Jurkat (B), or Z138 (C) lysate (n = 3 for BJAB and Jurkat, n = 6 for Z138; error bars are SEM). (D) Each DISC component was assigned to a category (receptors, FADD or DED-only) and the NSAF values combined. Total NSAF values were corrected to FADD to produce the stoichiometry of the TRAIL DISC from total cellular lysates. (E) Relative total levels of core DISC components in BJAB, Jurkat, and Z138 cells. Equivalent quantities of total cellular protein were subjected to SDS-PAGE and analyzed for core DISC components by western blotting. GAPDH served as loading control.

Mass spectrometry is primarily used to identify proteins but is increasingly used to quantitate the relative abundance of individual proteins present within protein complexes (Blondeau et al., 2004; Paoletti et al., 2006). Therefore, label-free quantitation, using the normalized spectral abundance factor (NSAF) for each component, was used to determine the relative stoichiometry of TRAIL DISC components in all three cell lines (Figures 3A–3D). TRAIL-R2 was the predominant receptor identified in the Jurkat TRAIL DISC (Figure 3B), whereas TRAIL DISC isolated from BJAB and Z138 cells (which express both TRAIL-R1 and TRAIL-R2; Figure 3E) contained more TRAIL-R1 than TRAIL-R2, suggesting that in these cells TRAIL-R1 is the predominant signaling receptor activated by soluble TRAIL (Figures 3A and 3C). The mass spectrometry data thus agree with our earlier studies, showing that primary cells from patients with hematological malignancies signal almost exclusively through TRAIL-R1 (MacFarlane et al., 2005a, 2005b).

Unlike caspase-8, the role of caspase-10 in the DISC is controversial (Kischkel et al., 2001; Sprick et al., 2002); in our study, caspase-10 was much less abundant than caspase-8 and was only detected by mass spectrometry in BJAB cells, suggesting that it does not have an obligatory role in TRAIL DISC signaling (Figure 3A). Another DED-only protein, c-FLIP_L_, was also present in very low amounts and was identified by LC-MS/MS and western blotting (data not shown) migrating at a molecular weight consistent with it being cleaved c-FLIP_L_ (p43). Intriguingly, c-FLIP has been reported as a dual regulator of caspase-8 activation and CD95-mediated apoptosis (Chang et al., 2002); furthermore, it has been suggested that c-FLIP may be essential for establishing CD95 threshold behavior (Lavrik et al., 2007; Schleich et al., 2012, in this issue of *Molecular Cell*). Thus, while the small amount of cleaved c-FLIP_L_ detected in our study is entirely consistent with the isolation of a fully activated TRAIL DISC, it is currently unknown whether, under low TRAIL activation regimes, c-FLIP plays any role in establishing threshold behavior.

Current ideas of DISC structure envisage a 1:1:1 ratio between death receptors, FADD, and caspase-8, but this stoichiometry does not explain how caspase-8 can be dimerized, which is essential for active site conformation and catalytic activation (Boatright et al., 2003; Donepudi et al., 2003; Hughes et al., 2009; Oberst et al., 2010). Importantly, our mass spectrometry data did not support a 1:1 stoichiometry between core components of the native TRAIL DISC isolated from BJAB, Z138, and Jurkat cells (Figures 3A–3C). Strikingly, FADD (the only known adaptor protein that directly links death receptors to DED-only proteins) was one of the least abundant proteins in the TRAIL DISC. The deviation from the proposed 1:1 stoichiometry for all DISC components was even more striking when these components were grouped together according to how they are recruited to the complex (Figure 3D). Calculating the NSAF value for each group of components (TRAIL-R or DED-only proteins) relative to the NSAF for FADD indicated that for each FADD molecule there were between two and five receptors and approximately four DED-only proteins (Figure 3D). Thus, DDs from multiple receptors may be required for efficient FADD recruitment. Importantly, a substoichiometric ratio of FADD to DED-only proteins has also been independently detected in the CD95 DISC (Schleich et al., 2012).

**Figure 4:**
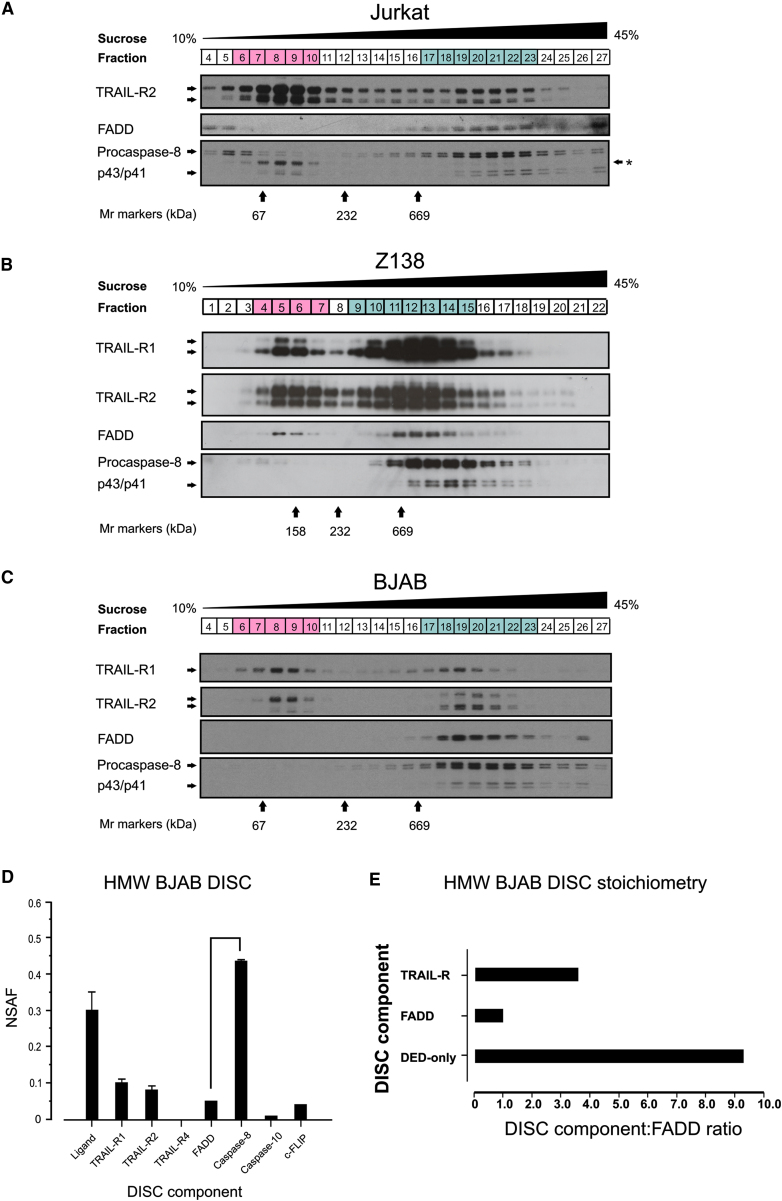
A High Molecular Weight TRAIL DISC Is Formed in BJAB, Jurkat, and Z138 Cells (A–C) Jurkat (A), Z138 (B), or BJAB (C) cells were treated with bTRAIL for 1 hr at 4°C and further incubated for 10 (BJAB) or 25 (Jurkat and Z138) min at 37°C. DISC was isolated from each fraction of a continuous 10%–45% sucrose density gradient. Fractions highlighted in pink contain low molecular weight ligand-receptor complexes, while blue fractions contain high molecular weight (HMW) DISC. Asterisk denotes residual signal from TRAIL-R2 antibody. Blots shown are representative of n = 3 (BJAB and Z138) or n = 2 (Jurkat). Molecular weight (Mr) markers (kDa) are indicated (n = 3). (D) Normalized spectral abundance factor (NSAF) analysis of LC-MS/MS data obtained for the HMW BJAB TRAIL DISC (Figure 4C, fractions 14–27) (n = 2; error bars, range). (E) Combined NSAF values for HMW DISC components corrected to FADD as in Figure 3D.

These stoichiometries were determined by spectral counting of TRAIL DISCs isolated from total cellular lysates. In this situation, it is possible that the stoichiometry of the TRAIL DISC may be skewed by the presence of partially assembled ligand-associated TRAIL-R complexes. Therefore, we used sucrose density gradient centrifugation to fractionate cell lysates into low molecular weight (LMW) ligand-associated complexes and high molecular weight (HMW) TRAIL DISC (Figure 1A, Expt B). Affinity purification of biotin-labeled TRAIL (and associated proteins) from sucrose density gradient fractions of cellular lysates revealed that a HMW DISC (>700 kDa) was formed in all three tumor cell lines (Figures 4A–4C). Sucrose density centrifugation successfully separated LMW ligand-bound TRAIL-Rs (Jurkat and BJAB cells, Figures 4A and 4C) or LMW subcomplexes of ligand, receptor, and FADD (Z138 cells, Figure 4B) from fully formed DISC. The relative proportions of LMW:HMW complexes varied according to cell type, with Jurkat cells containing predominantly LMW TRAIL-R complexes (Figure 4A, fractions 6–10), whereas Z138 and BJAB cells contained equivalent amounts of LMW TRAIL-R complexes and HMW DISC (Figures 4B and 4C). Thus, the ratio of receptor to FADD calculated for the Jurkat DISC isolated from total cell lysates (Figure 3) is likely to be skewed, overestimating the TRAIL-R2:FADD ratio. However, when we analyzed TRAIL DISC stoichiometry in the HMW DISC isolated from BJAB cells (Figure 4D; Figure S2B), we confirmed that FADD is substoichiometric compared to both TRAIL-Rs and caspase-8. Similar to our earlier stoichiometric calculations (Figure 3D), there was approximately 3-fold more TRAIL receptor than FADD in the HMW TRAIL DISC (Figure 4E). In view of the reproducibility of the ∼3:1 ratio between TRAIL receptors and FADD, the commonly held view of one death receptor recruiting one FADD molecule should now be re-evaluated.

### Structural Modeling Reveals that FADD Can Recruit Multiple DED-Only Proteins

Strikingly, quantitative mass spectrometry highlighted the difference in abundance between FADD and TRAIL-Rs or caspase-8, with the FADD:DED-only protein ratio increasing to 1:9 in the HMW TRAIL DISC (Figure 4D). The consistent identification of more caspase-8/DED-only proteins than FADD in the TRAIL DISC isolated from all three cell lines (Figure 3; Figures 4D and 4E), and the absence of any other molecule that could recruit DED-only proteins, led us to ask whether it was structurally feasible for FADD to recruit multiple DED-only proteins. We hypothesized that if only one of the two DEDs of caspase-8 was required for binding to FADD, could the other DED of caspase-8 potentially recruit additional DED-only proteins through a DED-DED interaction? Indeed, earlier studies have shown that the DEDs of caspase-8, caspase-10, and c-FLIP are able to interact with each other (Irmler et al., 1997; Wang et al., 2001) and that, upon transient overexpression, DEDs (from caspase-8 or FADD) can interact to form fibers known as death effector filaments (Barbero et al., 2008; Siegel et al., 1998).

**Figure 5:**
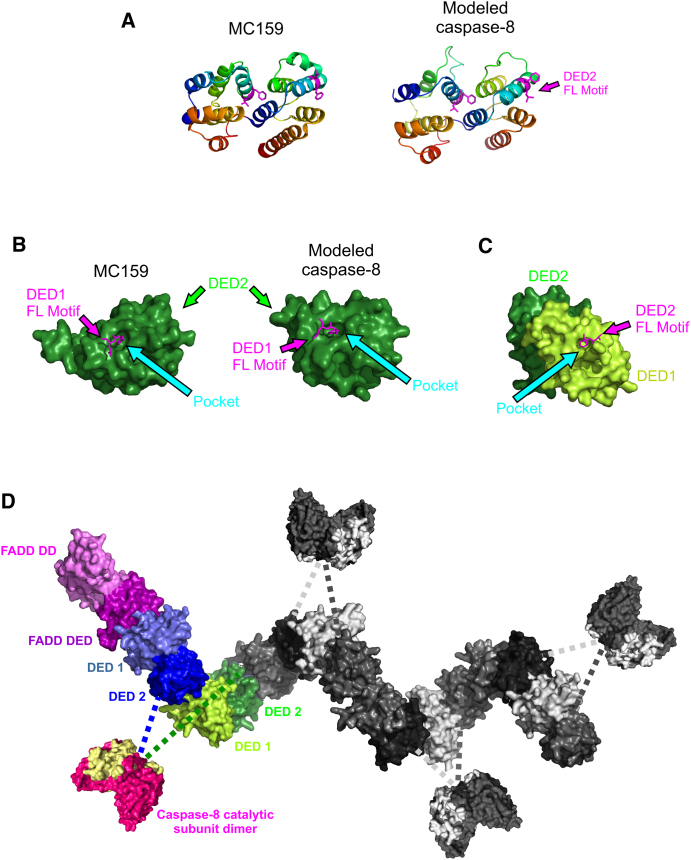
Structural Modeling of Procaspase-8 DED Chain Formation within the DISC (A) Published structure for MC159 used by Phyre to structurally model the DEDs of procaspase-8. (B) Surface structure of MC159 DED2 and modeled procaspase-8 DED2 showing the FL motif from DED1 interacting with a pocket on the surface of DED2. (C) Modeled surface structure of caspase-8 DEDs showing the pocket in DED1 that could interact with the FL motif from either FADD or DED2 of another molecule. The interaction interface between adjacent tandem DEDs was modeled using the intramolecular interface between DED1 and DED2. (D) Structural modeling of the interactions that may occur between the DEDs of FADD and caspase-8. Interactions between FADD (structure 2GF5) and procaspase-8 (or between adjacent sets of procaspase-8 DEDs) were modeled using the intramolecular interface between DED1 and DED2, resulting in a DED chain. The colored subunits of the model correspond to the model of the interaction between FADD DED and one dimer of procaspase-8. The extended chain is shown in gray. Catalytic subunit dimers (p18_2_/p10_2_, modeled from structure 3KJQ) are shown to indicate how we would expect antiparallel dimers to form along the length of the DED chain (dotted lines represent the linker between DED2 and p18 subunit of procaspase-8). Note that the helical character of the DED chain is uncertain, since small changes in the interface would significantly change the long-range topology.

Currently, structural information is not available for DED1/2 of caspase-8; therefore, we used the protein fold recognition server (Phyre) (Kelley and Sternberg, 2009) to construct a model based on the crystal structure of the viral FLIP, MC159 (Figure 5A) (Li et al., 2006; Yang et al., 2005). MC159 has previously been used to model interactions between DED1 and DED2 of caspase-8 and between caspase-8 DED2 and FADD (Carrington et al., 2006; Li et al., 2006; Yang et al., 2005). In MC159, DED1 interacts with DED2 via a phenylalanine/leucine (FL) hydrophobic motif (Figure 5A, magenta) that is conserved in nearly all DEDs. In tandem DED proteins (procaspase-8 and MC159), the DED1 “FL motif” occupies a pocket in the surface of DED2 (Figure 5B). Importantly, as FADD contains only one DED, the F25/L26 motif is not buried at an interface and thus is available to recruit procaspase-8. Indeed, mutation of this FL motif in FADD has been shown to prevent caspase-8 binding (Eberstadt et al., 1998). Although it has been suggested that procaspase-8 DED2 mediates recruitment to FADD (Yang et al., 2005), we reasoned that based on the DED1-DED2 interaction interface in MC159/procaspase-8, the FL motif of FADD could recruit procaspase-8 via its DED1 pocket (Figure 5C). Subsequently, this molecule of procaspase-8 would act as a scaffold and use its exposed DED2 F122/L123 motif to recruit an additional molecule of procaspase-8 via its DED1 pocket, which in turn could recruit additional procaspase-8 molecules (Figure 5C). To test the structural feasibility of this chain assembly model, we used the DED1-DED2 interaction interface to model the interaction between FADD and procaspase-8 DED1 and between procaspase-8 DED2 and the DED1 of additional molecules of procaspase-8. The resulting model would explain the stoichiometry determined by LC-MS/MS (Figures 3D and 4E), since multiple procaspase-8 molecules could be recruited to FADD and by self-association form a DED chain. Figure 5D depicts a potential configuration for the assembly of the DED chain. However, it should be noted that the exact long-range topology remains uncertain. Structural modeling also allowed us to approximate the orientation of the catalytic subunits (p18_2_/p10_2_) of caspase-8 in relation to their respective DEDs. Crucially, the DED chain model facilitates proximity-induced antiparallel dimerization of adjacent caspase-8 catalytic subunits (Boatright et al., 2003; Donepudi et al., 2003; Hughes et al., 2009). Thus, active dimers would lie along the length of the chain, orientated so that each active site is accessible to downstream DISC substrates that in turn trigger cell death.

### Mutation of DED2 FL Motif Abrogates DED Chain Formation and Procaspase-8 Activation

**Figure 6:**
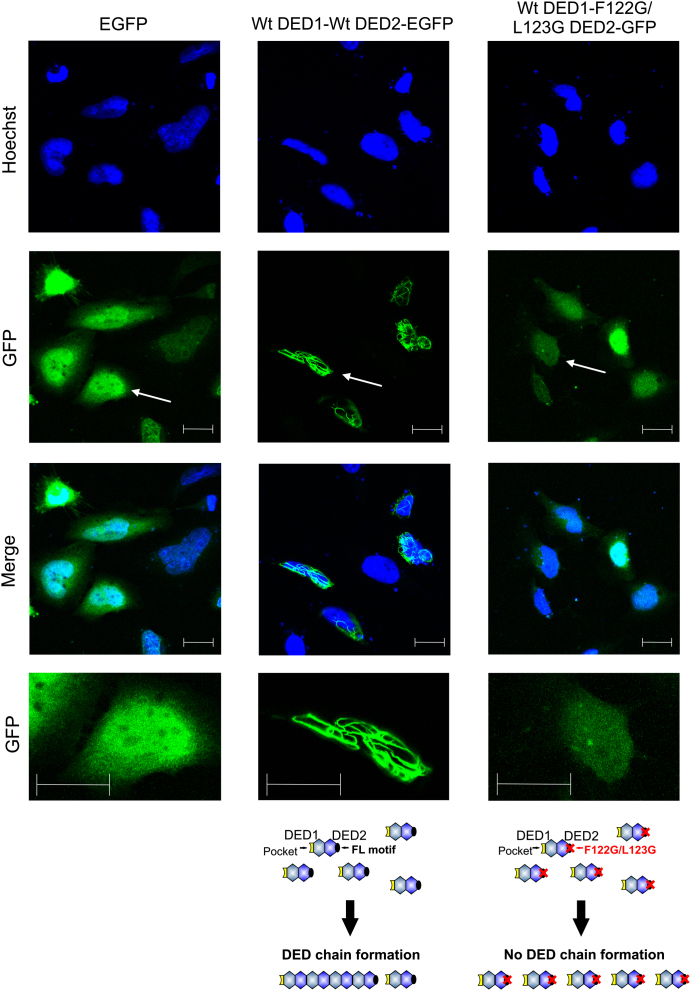
Mutation of the FL Motif in Caspase-8 DED2 Prevents Death Effector Filament Formation HeLa cells were transfected with either empty vector (EGFP) or the caspase-8 DED variants DED1-DED2-EGFP or DED1-DED2 F122G/L123G-EGFP for 20 hr before fixing and staining with Hoechst. Cells were imaged using a Zeiss LSM510 confocal microscope, and a representative field for each transfection is shown. Lower panels show enlargement of those areas arrowed in the GFP panels. Scale bar, 20 μm.

**Figure 7:**
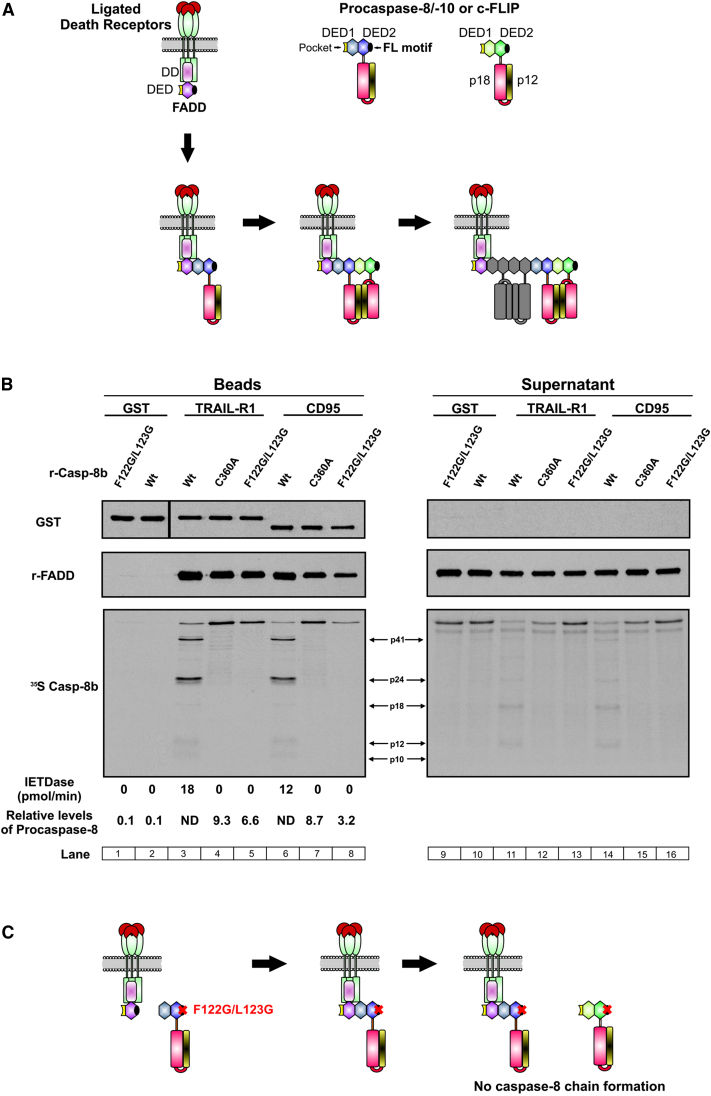
A Paradigm-Changing Model for Recruitment of Procaspase-8 and Other DED-Only Proteins to the DISC (A) Upon stimulation by the appropriate death ligand, death receptors recruit FADD via their death domains (DDs). FADD in turn recruits procaspase-8 (or procaspase-10 or c-FLIP) through a DED-mediated interaction. FADD may initially recruit one molecule of procaspase-8 via a single interaction with DED1 of procaspase-8 (for other permutations of this model, see Figure S4). The exposed DED2 FL motif then recruits additional molecules to produce a chain, facilitating dimerization and full activation of caspase-8/10. (B) Mutation of the DED2 FL motif reduces procaspase-8 recruitment and prevents processing in the in vitro DISC reconstitution model. Reconstituted DISC was formed with GST-CD95-IcD or GST-TRAIL-R1-ICD; recombinant FADD; and ^35^S-labeled IVT wild-type (WT), active site mutant (C360A), or F122G/L123G mutant caspase-8. Reconstituted DISC and supernatants were assessed for GST (loading control), ^35^S-labeled caspase-8, FADD, and IETDase activity. The relative levels of procaspase-8 recruited to the complex were determined by densitometry (corrected relative to GST-receptor-IcD). (C) Model depicting the potential impact of mutating the FL motif in procaspase-8 DED2. F122G/L123G does not prevent binding of caspase-8 to FADD but limits recruitment of additional procaspase-8 molecules, thus preventing DED chain formation and dimerization/activation of procaspase-8 catalytic subunits within the DISC.

In our structural-based DISC model, DED1 of procaspase-8 mediates recruitment to FADD, while DED2 mediates chain formation (Figure 5D; Figure 7A; Figure S4A, model 1). Notably, in procaspase-8 the DED2 FL motif is vital for DED chain formation, since mutation of both residues to glycine (F122G/L123G) disrupts death effector filament formation upon transient overexpression of DEDs (Figure 6). However, there are three alternative variations of the model in Figure 5D. Either procapase-8 DED2 interacts with FADD while DED1 mediates chain formation (Figure S4A, model 2); alternatively, two procaspase-8 molecules could be recruited simultaneously to FADD, one by DED1 and the other by DED2, with chain formation mediated via DED2 or DED1, respectively (Figure S4A, model 3). Finally, FADD from different receptor complexes could self-associate, and then caspase-8 chain formation could proceed simultaneously from each FADD molecule (Figure S4A, model 4).

To provide direct experimental proof for this caspase-8 DED chain structure and distinguish between the alternative models, we employed an in vitro reconstituted DISC using full-length recombinant proteins (Hughes et al., 2009). This allowed us to examine procaspase-8 recruitment and activation within the DISC upon mutation of DED2 FL motif (Figures 7B and 7C). Mutation of DED2 FL motif (F122G/L123G) significantly reduced procaspase-8 recruitment to either CD95 or TRAIL-R1 DISC, as compared to the unprocessed active site mutant (C360A) (Figure 7B, compare lanes 4 and 5, and lanes 7 and 8). Furthermore, the F122G/L123G mutation abolished procaspase-8 activation within the complex, as shown by the absence of autoprocessing and inability to cleave IETD-AFC (Figure 7B, compare lanes 3 and 5, and lanes 6 and 8). Since mutation of the FL motif in DED2 reduced but did not totally prevent procaspase-8 recruitment, it is unlikely that procaspase-8 is solely recruited to FADD via DED2 (Figures S4A and S4B, model 2). Thus, only the models in which procaspase-8 is recruited to FADD by DED1 (Figure 5D, Figure 7A; Figures S4A and S4B, model 1) or simultaneously by DED1 and DED2 (Figures S4A and S4B, models 3 and 4) are feasible. Crucially, the in vitro reconstituted DISC model provides direct proof of principle for caspase-8 DED-mediated chain formation within the DISC.

## Discussion

DISC formation is the key initiating event of the extrinsic pathway, enabling activation of the caspase cascade that results in apoptotic cell death. Despite extensive research, our understanding of how this complex assembles and triggers caspase activation or alternative signaling pathways is incomplete. Using biochemical approaches and quantitative mass spectrometry, we have isolated and characterized the TRAIL DISC proteome from BJAB, Z138, and Jurkat cells (Figure 1). In all three DISC preparations, mass spectrometry successfully identified the core components, TRAIL-R1/R2, FADD, and caspase-8; other proteins such as c-FLIP and caspase-10 were detected, but only in very small amounts. While putative DISC interactors would be predicted to be present in at least a 1:1 ratio with core components, additional proteins which have previously been reported in the literature (Harper et al., 2001; reviewed in Peter and Krammer, 2003) were not detected. Our consistent identification of only the core components suggests that several proteins previously suggested to be associated with the DISC (e.g., DAXX, FLASH, FAP-1, DAP3, and FAF-10) are either absent or in very low (undetectable) amounts in the native TRAIL DISC.

Several studies have suggested that CD95 and TRAIL-R1/R2 require LRs for effective apoptotic signaling (Muppidi and Siegel, 2004; Rossin et al., 2009; Song et al., 2007), but very few studies have established colocalization of FADD, caspase-8, and TRAIL-Rs in LRs after TRAIL treatment. Our analysis of the TRAIL DISC in hematopoeitic cells showed that only small amounts (<5%) of the DISC is associated with LRs and that the majority of the complex is soluble (Figure 2C). These data agree with similar studies on CD95 (Feig et al., 2007). However, LRs may play a greater role in TRAIL signaling in epithelial tumor cells (e.g., NSCLC) (Song et al., 2007), and this likely explains why we only detected cullin-3, a LR-associated protein, in the HeLa TRAIL DISC (Figure 2A). Therefore, the role of LRs, and hence the proteomic composition of the DISC, may vary according to cell type.

Quantitative mass spectrometry allowed us to determine the stoichiometry of core DISC proteins, and surprisingly this did not fit with current thinking, which is based on a 1:1:1 ratio between TRAIL-R1/R2, FADD and caspase-8. However, prevailing ideas on DISC stoichiometry are hampered by the fact that these are largely based on in vitro findings with truncated (domain-depleted) recombinant proteins, which may not truly replicate DISC oligomeric structure/function when full-length native proteins are involved. With truncated proteins there appears to be a 1:1 ratio between the DDs of CD95 and FADD (Esposito et al., 2010; Scott et al., 2009; Wang et al., 2010). In our study, the receptor:FADD ratio in the native DISC from Z138/BJAB cells was instead ∼3:1 (Figures 3D and 4E), suggesting that more than one receptor may be required for efficient FADD recruitment. Indeed, our finding of an ∼3:1 ratio of death receptor to FADD in the native TRAIL DISC is entirely consistent with there being a requirement for receptor clustering in order to overcome the signaling threshold for receptor activation. Furthermore, the observation that membrane-bound CD95L (and by extension also membrane-bound TRAIL), but not the soluble form, is critical for apoptosis (O'Reilly et al., 2009) raises the intriguing possibility that binding of membrane-bound ligand results in formation of a more stable complex which in turn may enhance caspase-8 recruitment/DED chain assembly at the DISC.

The higher than expected ratio of DED-only proteins to FADD indicated that multiples of procaspase-8 must be recruited to the DISC via a single FADD. Interestingly, the ratio of DED-only proteins recruited to FADD varied between the three cell lines (Figure 3). Thus, it may be that DED chain length varies between cell types, and/or that DED chain formation is highly dynamic, dependent on the time and strength of the death ligand signal and/or the availability of DISC components. In BJAB cells, the caspase-8:FADD ratio increased 2-fold when the TRAIL HMW DISC was gradient purified (Figure 4D). This increased caspase-8:FADD ratio partly reflects the removal of LMW ligand-associated TRAIL-R complexes, and the result of better identification of DED-only proteins by mass spectrometry in the highly purified DISC where ion suppression and/or peptide competition for MS acquisition are minimized. Strikingly, in the TRAIL HMW DISC, DED-only proteins were on average approximately nine times more abundant than FADD (Figure 4E).

The concept of high DED-only:FADD ratios clearly challenges existing paradigms, and while NSAF values can provide a good indication as to the relative amounts of components within the complex, they are not an absolute quantitation. It was therefore critical that other independent approaches were employed to validate this paradigm-changing DISC model. Structural modeling tested the feasibility of DED-dependent caspase-8 chain formation and provided four models for procaspase-8 recruitment to the DISC, which varied in the precise DED domain responsible for the initial recruitment to FADD but possessed the common theme of DED chain formation. Current models suggest that only one DED (DED2) is responsible for tandem DED protein recruitment to the DISC (Yang et al., 2005). However, this does not explain why procaspase-8/procaspase-10 and c-FLIP have two DED domains, and why both are apparently required for successful recruitment to the complex (Tsukumo and Yonehara, 1999; Yao et al., 2007). Importantly, our structural-based DISC model now offers a functional explanation for the presence of two DEDs; thus, one DED mediates recruitment to FADD, while the other DED recruits the next procaspase-8 molecule in the DED chain.

Significantly, the DEDs of c-FLIP, caspase-8, and caspase-10 have been shown to associate with themselves and each other (Irmler et al., 1997; Wang et al., 2001), and filament formation has been observed during transient overexpression of DEDs (Barbero et al., 2008; Siegel et al., 1998). Caspase-8 DED filament formation was abrogated through selective mutation of DED2 FL motif (F122G/L123G) (Figure 6). This provides further evidence for our DED chain hypothesis but also suggests that the FL motif in DED2 is crucial for procaspase-8 DED chain elongation at the DISC (Figure S4). Importantly, mere overexpression of full-length caspase-8 does not induce filament formation (Barbero et al., 2008; MacFarlane et al., 2000; Siegel et al., 1998), suggesting that the catalytic subunits of procaspase-8 shield the DED interaction sites. Thus, for DISC-mediated DED chain formation to occur, a structural rearrangement of procaspase-8, triggered by death receptor/FADD ligation and/or an increase in their local concentration, would be required to unmask key DED interaction sites. Furthermore, there is also the intriguing possibility that proteins of the cytoskeleton may play a role in triggering DED chain formation, particularly in light of several reports suggesting a context-dependent role for proteins of the cytoskeleton in triggering CD95-induced apoptosis (reviewed in Chaigne-Delalande et al., 2008).

Four structurally viable models of procaspase-8 recruitment to the DISC explain how either DED1 or DED2 could be responsible for the initial recruitment of procaspase-8 to FADD (Figure 7A and Figure S4). Thus, DED1 could interact with the FL motif of FADD, while in a DED2-mediated interaction the DED2 FL motif would bind to FADD. Both scenarios are supported by previous mutation studies, with mutation of either F25 in FADD (Eberstadt et al., 1998) or F122/L123 of procaspase-8 (DED2 FL motif) preventing FADD-procaspase-8 interaction (Yang et al., 2005). However, in contrast to Yang et al., who reported that mutation of DED2 FL motif prevented FADD-procaspase-8 coimmunoprecipitation following their overexpression (Yang et al., 2005), we show that mutation of DED2 FL motif (F122G/L123G) interferes with, but does not totally prevent, procaspase-8 recruitment to FADD within the CD95/TRAIL-R1 DISC. Our data, using full-length recombinant proteins in a reconstituted DISC, favors either model 1 or model 3/4 in which DED2 mutation would not completely abrogate caspase-8 recruitment (Figure 7C and Figure S4B). In model 1, mutation of DED2 would prevent chain formation but not initial recruitment of procaspase-8 to FADD via DED1. By preventing the stacking of adjacent procaspase-8 molecules, the DED2 FL motif mutant would block formation of the DED chain and hence the correct alignment of the catalytic dimers (p18_2_/p10_2_) that lie along it (Figure 5D). This scenario fits perfectly with the inactive nature of the DED2 FL motif mutant in the in vitro reconstituted DISC. Taken together, these data provide direct experimental evidence in support of our DED chain assembly model, whereby procaspase-8 is recruited to FADD and through DED-mediated structural rearrangement forms catalytically active caspase-8 chains.

We have previously shown that the in vitro reconstituted DISC model can accurately mimic the cellular response to death ligands (Hughes et al., 2009). Since DED2 F122G/L123G mutant procaspase-8 is catalytically inactive in both the CD95 and TRAIL-R1 reconstituted DISC, we predict that disruption of DED chains would prevent cleavage of downstream effectors, thereby impeding apoptotic cell death (Hughes et al., 2009 and data not shown). Our DED chain assembly model is further supported by the observation that FADD-DN mutants, previously shown to have a profound effect on apoptosis, would be predicted to inhibit caspase-8 chain elongation (Newton et al., 1998). Importantly, this could enable a shift toward activation of alternative modes of cell death/signaling pathways where the catalytic activity of procaspase-8 is not required (Oberst et al., 2011). Interestingly, several procaspase-8 DED mutations have been identified in gastric and colon carcinomas (Kim et al., 2003; Soung et al., 2005); crucially, these mutations may prevent DED chain formation and cell death, thus promoting inappropriate cell survival. Furthermore, DED chain formation may not be limited to membrane-associated receptor complexes and may also play an important role in the activation of procaspase-8 in soluble complexes, such as the RIG-1 complex which modulates immune response (Rajput et al., 2011). Overall, the propensity to form DED chains likely affects the critical balance between cell death or activation of alternative signaling pathways, and as such may play a major role in determining cell fate.

In summary, we propose a paradigm-changing model for DISC assembly and structure whereby FADD is substoichiometric and procaspase-8 is recruited, not only through an interaction with FADD but also by interacting with itself. The DED chain assembly model also presents the intriguing possibility that only a small amount of DISC would be required to activate large amounts of caspase-8. This amplification phenomenon could be hugely important in the context of normal cell physiology and disease, as it is now clear that caspase-8 activation needs to be tightly regulated not only at the DISC but in several other signaling platforms in order to prevent inappropriate cell death/activation of alternative signaling pathways.

## Experimental Procedures

### Cell Lines

The cell lines BJAB (Burkitt's lymphoma), Jurkat (T cell leukemia), and Z138 (mantle cell lymphoma) were obtained and cultured in RPMI (supplemented with 10% FCS 1% Glutamax) and maintained in 5% CO_2_ at 37°C as previously described (Hughes et al., 2009).

### Affinity Purification of TRAIL DISC

Biotin-labeled TRAIL (bTRAIL) and Strep-II-tagged TRAIL (stTRAIL) (Inoue et al., 2009) were generated and affinity purification of the TRAIL DISC performed as previously described (Harper and MacFarlane, 2008), with the following modifications. Cells were incubated on ice for 1 hr with 500 ng/ml of tagged TRAIL, followed by further incubation for 10 (BJAB) or 25 (Jurkat, Z138) min at 37°C. Unbound ligand was removed by PBS washes and the cells lysed on ice in DISC lysis buffer (30 mM Tris-HCl [pH 7.5], 150 mM NaCl, 10% glycerol, 1% Triton X-100, and protease inhibitor tablets). In total DISC pull-downs, the resulting cleared lysate (Figure S1) was incubated with streptavidin/streptactin beads for ligand complex capture (Figure 1A, Expt A). In gradient pull-downs, the cleared lysate was subjected to fractionation by 10%–45% linear sucrose density gradients or LR flotation, prior to the addition of beads (Figure 1A, Expt B and Expt C, respectively). LRs were prepared as previously described (Boyd et al., 2009), and proteins were concentrated by chloroform/methanol precipitation or by ultracentrifugation (1 hr at 100,000 × g). Linear sucrose density gradients (50 mM Tris-HCl [pH 7.4], 150 mM NaCl, 0.1% Triton X-100, 10%–45% sucrose) were generated and cleared cell lysate or molecular weight markers sedimented by centrifugation (100,000 × g for 17 hr at 4°C) into 0.5 ml fractions. TRAIL DISC was eluted and solubilized with 1× SDS sample buffer at 95°C. SDS samples were separated by SDS-PAGE before analysis by either mass spectrometry or western blotting.

### Western Blotting

Western blotting was performed as previously described (Harper and MacFarlane, 2008). Antibodies used were as follows: anti-Lyn, anti-Flotillin 1, and anti-FADD (BD Biosciences), anti-TRAIL-R1 (ProSci Inc.), anti-TRAIL-R2 (Cell Signaling Technology), anti-cullin-3 (Abcam), anti-strep II tag (IBA GmbH), anti-caspase-10 (MBL), anti-c-FLIP (NF6; Enzo Life Sciences), anti-GAPDH (Advanced Immunochemical Inc.) and anti-caspase-8.

### Mass Spectrometry

Purified TRAIL complexes were analyzed by shotgun proteomics as previously described (Boyd et al., 2009). Briefly, SDS-PAGE gels were sliced, destained, dehydrated, and digested with trypsin (Promega) either overnight at 30°C or for 3 hr at 37°C. Tryptic peptides were extracted using 0.2% trifluoroacetic acid, solubilized in 5% formic acid, and analyzed by LC-MS/MS using a nanoLC system (CapLC, Waters) interfaced to a QTof hybrid mass spectrometer (Waters). Mass spectrometry data were acquired using Masslynx 4.0 with automatic precursor ion selection for double and highly charged ions. Peptide spectra were processed by Proteinlynx software identified though comparison to the nonredundant SwissProt database (ftp://us.expasy.org/databases/) using the MASCOT program (Matrix Science). Peptide probabilities were calculated using the algorithm “Peptide Prophet,” which converts the output from the Mascot search engine into a discriminant score (Nesvizhskii et al., 2003). The algorithm, “Protein Prophet,” uses the same output to estimate protein identification probabilities (Keller et al., 2002). Protein and peptide identification were validated using Scaffold (Proteome Software Inc.).

### Analysis of Mass Spectrometry Data

Proteins identified by mass spectrometry were further analyzed according to the following criteria: the protein was identified with 95% peptide and 50% protein probabilities and with convincing spectra but was not identified in the control sample (unless in dramatically lower abundance than in the treated sample), was not a known contaminant (e.g., carboxylases which require biotin as a cofactor) or previously identified to nonspecifically interact with purification beads (Trinkle-Mulcahy et al., 2008), and had a mascot ion score of greater than 30 and did not have a large parent ion mass error. The only deviation from this was to include the identification of FADD in the Jurkat TRAIL DISC. SAF and NSAF were calculated as previously described (Blondeau et al., 2004; Paoletti et al., 2006). NSAF values were calculated using the spectral counts with 50% protein and peptide probability thresholds, and for the HMW BJAB TRAIL DISC, spectral counts were from MudPit analysis of the mass spectrometer data sets.

### Transfection and Visualization of Death Effector Filaments

HeLa cells were seeded onto coverslips in 24-well plates 1 day prior to transfection. Vector DNA (0.5 μg) was transfected into cells using Lipofectamine 2000 (Invitrogen) according to the manufacturer's instructions. Twenty hours after transfection, cells were fixed in Accustain Formalin solution (Sigma) and the nuclei stained using Hoechst 33342 (Molecular Probes) (MacFarlane et al., 2000). Coverslips were mounted onto slides and multiple fields imaged with a LSM510 confocal microscope (Zeiss). Image processing was performed with Zen 2009 (Zeiss).

### CD95 and TRAIL-R1 DISC Reconstitutions

In vitro DISC reconstitutions were completed as previously described (Hughes et al., 2009). Briefly, 10 μg of purified GST-CD95-IcD or GST-TRAIL-R1-IcD fusions (bound to glutathione Sepharose beads) were incubated with recombinant FADD (5 μg) and IVT-generated ^35^S-labeled procaspase-8 (wild-type, C360A, or F122G/L123G) for 16 hr at 23°C. Bead-associated complexes were eluted by boiling in SDS sample buffer and analyzed by western blotting. The proteolytic activity of the bead-bound complex was assessed fluorometrically with Ac-IETD.AFC.

### Structural Modeling of Procaspase-8 Recruitment to FADD

The modeled structure of the DEDs of caspase-8 was generated by Phyre (Kelley and Sternberg, 2009). Amino acids 1–183 of caspase-8 (Q14790) were submitted to the Phyre server, and the structure resulting from the threading of this sequence through the published structure of MC159 (2BBR) was selected as our modeled structure of caspase-8 DED1/2. The DED domains of caspase-8 and FADD (structure 2GF5) were aligned using LSQman (Kleywegt, 1996) to determine which regions of the DED domains were similar (rmsd of less than 3). PyMOL (http://www.pymol.org/) was then used to generate a caspase-8 DED chain. The interactions between DEDs from different procaspase-8 molecules were modeled using the intramolecular interface that exists between DED1 and DED2 in the tandem DEDs of the modeled caspase-8 structure. As it stands, the model suggests that the DED chain would have a helical character, but this cannot be confidently predicted, since small changes at the DED interface would have a significant impact on the long-range topology of the chain. To reflect this uncertainty, we have colored the distal DEDs in gray.
