# Supplementary material for: A Death Effector Domain Chain DISC Model Reveals a Crucial Role for Caspase-8 Chain Assembly in Mediating Apoptotic Cell Death
Source: Mol Cell. 2012 Jul 27;47(2-2):291–305. doi: 10.1016/j.molcel.2012.05.004 (PMC3477315; doi:10.1016/j.molcel.2012.05.004)
Supplement: Document S1. Figure S1, Figure S2, Figure S3, and Figure S4 [file mmc1.pdf]

Molecular Cell, Volume *47*

Supplemental Information

A Death Effector Domain Chain DISC Model Reveals a Crucial Role for Caspase-8 Chain Assembly in Mediating Apoptotic Cell Death

Laura S. Dickens, Robert S. Boyd, Rebekah Jukes-Jones, Michelle A. Hughes, Gemma L. Robinson, Louise Fairall, John W.R. Schwabe, Kelvin Cain, and Marion MacFarlane

Inventory of Supplemental Information

Figure S1, related to Fig. 1
TRAIL DISC components are exclusively localized in the cleared cell lysate

Figure S2, related to Fig. 1 and Fig. 4

Known components of the TRAIL DISC were identified by mass spectrometry by multiple peptides and spectra

Figure S3, related to Fig. 2

Lipid raft markers were not associated with soluble TRAIL DISC (S-DISC)

Figure S4, related to Fig. 5 and Fig. 7

Putative models for the recruitment of procaspase-8 and other DED-only proteins to the DISC

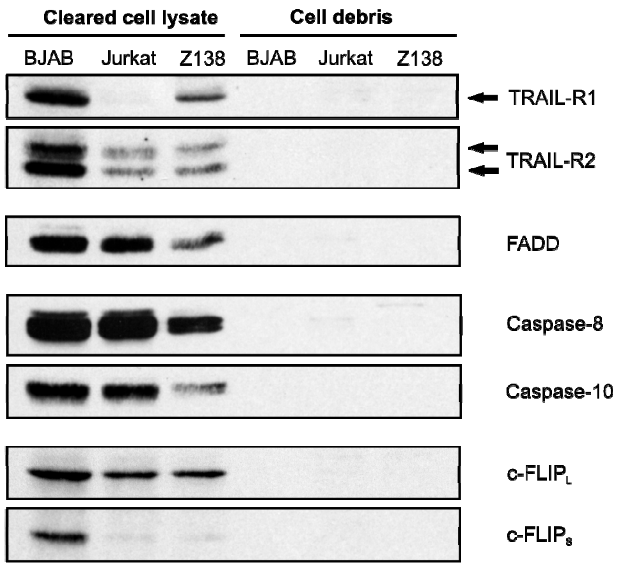

Figure S1. TRAIL DISC components are exclusively localized in the cleared cell lysate. BJAB, Jurkat or Z138 cells were stimulated with biotin-labelled (bTRAIL) as described in Experimental Procedures. Following TRAIL DISC formation and subsequent cell lysis, matched volumes of cleared cell lysate and cell debris were separated by SDS-PAGE and analyzed by western blotting for the presence of TRAIL-Rs, FADD, Caspase-8, Caspase-10 and c-FLIP($_L$ and $_S$). In matched samples of cleared cell lysate and cell debris, all core DISC components were exclusively localized in the cleared cell lysate across all three cell lines.

A

O00220 TR10A_HUMAN TRAIL-R1 - Homo sapiens (Human)

MAPPPARVHL GAFLAVTPNP GSAASGTEAA AATPSKVWGS SAGRIEPRGG
GRGALPTSMG QHGPSARARA GRAPGPRPAR EASPRLRVHK TFKFVVVGVL
LQVVPSSAAT IKLHDQSIGT QQWEHSPLGE LCPPGSHRSE HPGACNRCTE
GVGYTNASNN LFACLPCTAC KSDEEERSPC TTTR**NTACQC KPGTFR**NDNS
AEMCRKCSRG CPRGMVKVKD CTPWSDIECV HKESGNGHNI WVILVVTLVV
PLLLVAVLIV CCCIGSGCGG DPKCMDRVCF WRLGLLRGPG AEDNAHNEIL
SNADSLSTFV SEQQMESQEP ADLTGVTVQS PGEAQCLLGP AEAEGSQRRR
LLVPANGADP TETLMLFFDK FANIVPFDSW DQLMRQLDLT KNEIDVVRAG
TAGPGDALYA MLMKWVNKTG R**NASIHTLLD ALER**MEERHA R**EKIQDLLVD**
SGKFIYLEDG TGSAVSLE

O14763 TR10B_HUMAN TRAIL-R2 - Homo sapiens (Human)

MEQRGQNAPA ASGARKRHGP GPREARGARP GPRVPKTLVL VVAAVLLLVS
AESALITQQD LAPQQRAAPQ QKRSSPSEGL CPPGHHISED GRDCISCKYG
QDYSTHWNDL LFCLRCTR**CD SGEVELSPCT TTR**NTVCQCE EGTFREEDSP
EMCRKCRTGC PRGMVKVGDC TPWSDIECVH KESGTKHSGE VPAVEETVTS
SPGTPASPCS LSGIIIGVTV AAVVLIVAVF VCKSLLWKKV LPYLK**GICSG**
GGGDPERVDR SSQRPGAEDN VLNEIVSILQ PTQVPEQEME VQEPAEPTGV
NMLSPGESEH LLEPAEAERS QRRR**LLVPAN EGDPTETLR**Q CFDDFADLVP
FDSWEPLMR**K LGLMDNEIK**V AKAEAAGHR**D TLYTMLIK**WV NKTGR**DASVH**
TLLDALETLG ERLAKQKIED HLLSSGK**FMY LEGNADSAMS**

Q9UBN6 TR10D_HUMAN TRAIL-R4 - Homo sapiens (Human)

MGLWGQSVPT ASSARAGRYP GARTASGTRP WLLDPKILKF VVFIVAVLLP
VRVDSATIPR QDEVPQQTVA PQQQRRSLKE EECPAGSHRS EYTGACNPCT
EGVDYTIASN NLPSCLLCTV CKSGQTNKSS CTTTRDTVCQ CEKGSFQDKN
SPEMCRTCRT GCPRGMVKVS NCTPRSDIKC KNESAASSTG KTPAAEETVT
TILGMLASPY HYLIIIVVLV IILAVVVVGF SCRKKFISYL KGICSGGGGG
PERVHRVLFR RRSCPSRVPG AEDNARNETL SNRYLQPTQV SEQEIQGQEL
AELTGVTVES PEEPQRLLEQ AEAEGCQRRR **LLVPVNDADS ADISTLLDAS**
ATLEEGHAKE TIQDQLVGSE KLFYEEDEAG SATSCL

Q13158 FADD_HUMAN FADD - Homo sapiens (Human)

MDPFLVLLHS VSSSLSSSEL TELKFLCLGR VGKRKLER**VQ SGLDLFSMLL**
EQNDLEPGHT ELLRELLASL RRHDLLRRVD DFEAGAAAGA APGEEDLCAA
FNVICDNVGK DWRRLARQLK **VSDTKIDSIE DRYPR**NLTER VRESLRIWK**N**
TEKENATVAH LVGALRSCQM NLVADLVQEV QQARDLQNRS GAMSPMSWNS
DASTSEAS

Q14790 CASP8_HUMAN Caspase-8 - Homo sapiens (Human)

MDFSR**NLYDI GEQLDSEDLA SLKFLSLDYI PQRKQEPIKD ALMLFQR**LQE
KR**MLEESNLS FLK**ELLFR**IN RLDLLITYLN TR**KEEMEREL QTPGRAQISA
YR**VMLYQISE EVSR**SELRSF K**FLLQEEISK** CKLDDDMNLL DIFIEMEKRV
ILGEGKLDIL KRVCAQINKS LLK**IINDYEE FSK**ERSSSLE GSPDEFSNGE
ELCGVMTISD SPR**EQDSESQ TLDK**VYQMKS KPRGYCLIIN NHNFAKAREK
VPKLHSIRDR NGTHLDAGAL TTTFEELHFE IKPHDDCTVE QIYEILKIYQ
LMDHSNMDCF ICCILSHGDK **GIIYGTDGQE APIYELTSQF TGLK**CPSLAG
KPK**VFFIQAC QGDNYQK**GIP VETDSEEQPY LEMDLSSPQT RYIPDEADFL
LGMATVNNCV SYRNPAEGTW YIQSLCQSLR ERCPR**GDDIL TILTEVNYEV**
SNKDDKKNMG KQMPQPTFTL RKKLVFPSD

Q92851 CASPA_HUMAN Caspase-10 - Homo sapiens (Human)

MKSQGQHWYS SSDKNCKVSF REKLLIIDSN LGVQDVENLK FLCIGLVPNK
KLEKSSSASD VFEHLLAEDL LSEEDPFFLA ELLYIIRQKK LLQHLNCTKE
EVERLLPTRQ RVSLFR**NLLY ELSEGIDSEN LKDMIFLLK**D SLPK**TEMTSL**
SFLAFLEKQG KIDEDNLTCL EDLCKTVVPK LLRNIEKYKR EKAIQIVTPP
VDKEAESYQG EEELVSQTDV KTFLEALPQE SWQNKHAGSN GNRATNGAPS
LVSRGMQGAS ANTLNSETST KRAAVYRMNR NHRGLCVIVN NHSFTSLKDR
QGTHKDAEIL SHVFQWLGFT VHIHNNVTKV EMEMVLQKQK CNPAHADGDC
FVFCILTHGR **FGAVYSSDEA LIPIR**EIMSH FTALQCPRLA EKPKLFFIQA
CQGEEIQPSV SIEADALNPE QAPTSLQDSI PAEADFLLGL ATVPGYVSFR
HVEEGSWYIQ SLCNHLKKLV PRMLKFLEKT MEIRGRKRTV WGAKQISATS
LPTAISAQTP RPPMRRWSSV S

O15519 CFLAR_HUMAN c-FLIP - Homo sapiens (Human)

MSAEVIHQVE EALDTDEKEM LLFLCR**DVAI DVVPPNVRDL LDILR**ERGK**L**
SVGDLAELLY RVRRFDLLKR ILKMDRKAVE THLLRNPHLV SDYRVLMAEI
GEDLDKSDVS SLIFLMKDYM GRGKISKEK**S FLDLVVELEK** LNLVAPDQLD
LLEKCLKNIH RIDLKTKIQK YK**QSVQGAGT SYRNVLQAAI QK**SLKDPSNN
FRLHNGRSKE QRLKEQLGAQ QEPVKKSIQE SEAFLPQSIP EERYKMKSKP
LGICLIIDCI GNETELLR**DT FTSLGYEVQK** FLHLSMHGIS QILGQFACMP
EHR**DYDSFVC VLVSR**GGSQS VYGVDQTHSG LPLHHIRRMF MGDSCPYLAG
KPKMFFIQNY VVSEGQLEDS SLLEVDGPAM KNVEFKAQKR GLCTVHREAD
FFWSLCTADM SLLEQSHSSP SLYLQCLSQK LRQERKRPLL DLHIELNGYM
YDWNSRVSAK EKYYVWLQHT LRKKLILSYT

B

	High molecular weight BJAB TRAIL DISC	
	Experiment 1	Experiment 2
TRAIL	22	16
TRAIL-R1	18	8
TRAIL-R2	15	6
FADD	4	2
Caspase-8	81	40
Caspase-10	2	0
c-FLIP	7	0

Figure S2. Known components of the TRAIL DISC were identified by mass spectrometry by multiple peptides and spectra.

(A) Known components were identified by good peptide coverage. Peptides, corresponding to the known components of the TRAIL DISC, identified by mass spectrometry are depicted in bold. Red bold peptides were identified following mass spectrometry of the HMW BJAB TRAIL DISC (50 % peptide and protein probabilities, MudPit analysis). Black bold peptides are additional peptides that were identified by mass spectrometry of unfractionated TRAIL DISC isolated from BJAB, Jurkat or Z138 cells (50 % peptide and protein probabilities).

(B) Mass spectrometry of HMW weight BJAB TRAIL DISC resulted in the identification of multiple spectra for the known components. Numbers are the total assigned spectra for each known component across two experiments.

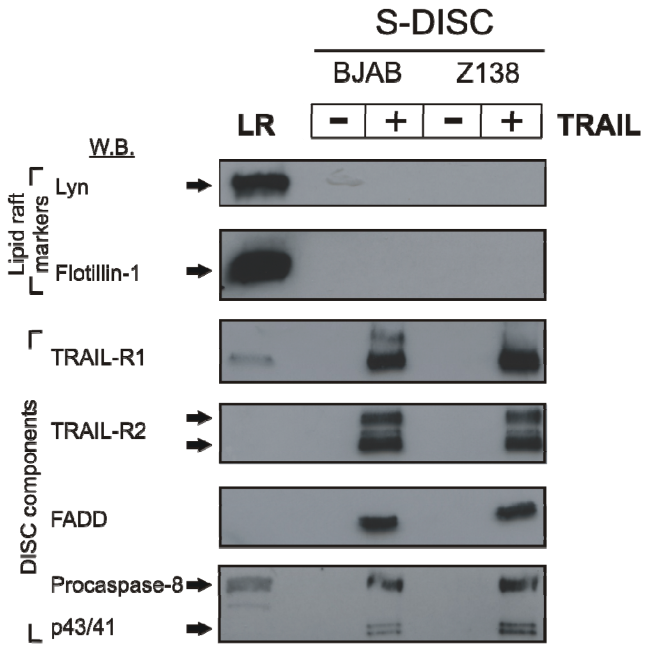

Figure S3 Lipid raft markers were not associated with soluble TRAIL DISC (S-DISC). Lipid raft markers are not associated with TRAIL DISC isolated from the soluble fraction of cells. TRAIL DISC was isolated from the soluble fraction of TRAIL treated BJAB or Z138 cells (S-DISC) and the presence of Lyn and Flotillin-1 (lipid raft markers) was investigated by western blotting. LR, Lipid raft fraction.

Figure S4

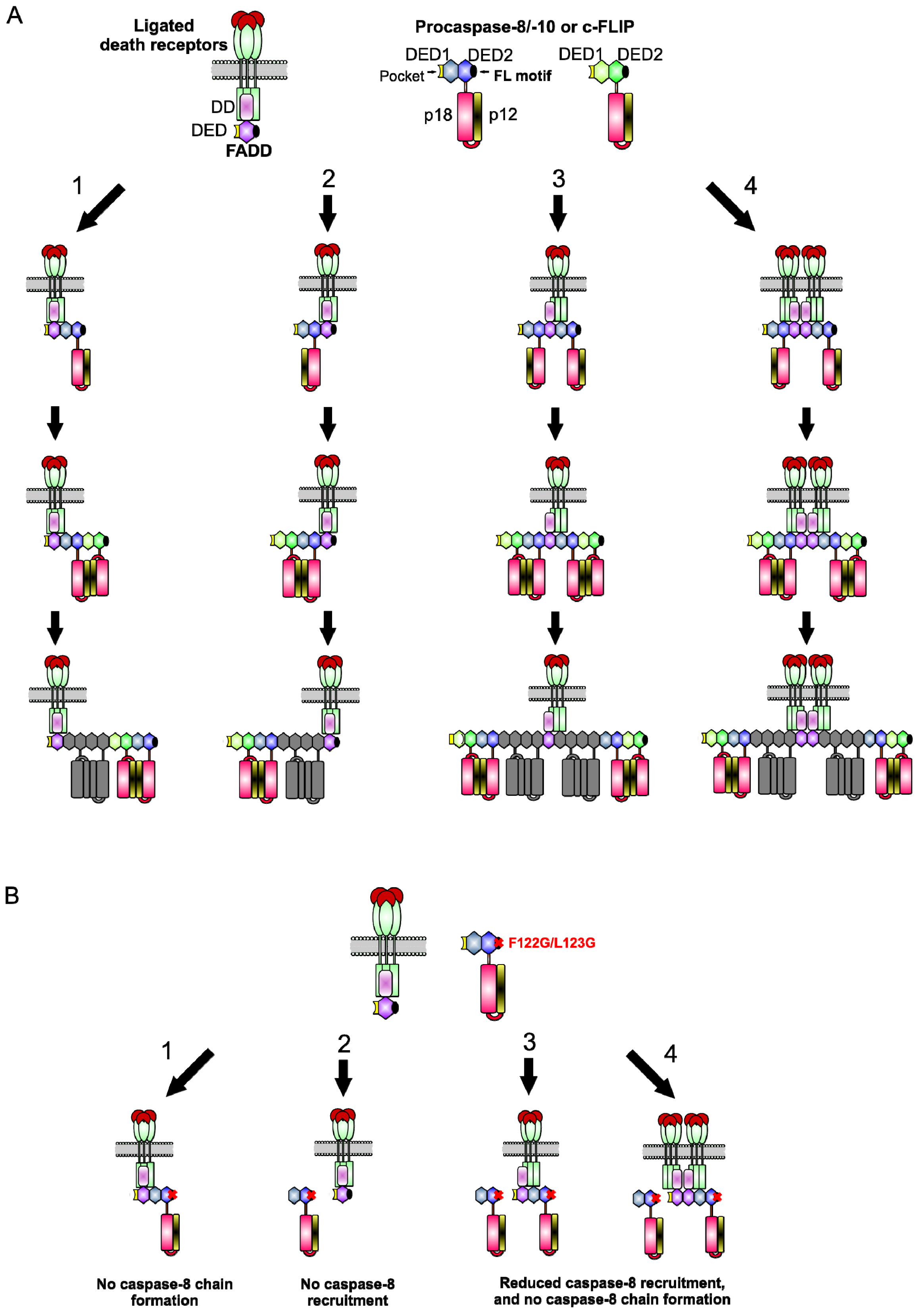
A
Ligated
death receptors
DD
DED
FADD
Procaspase-8/-10 or c-FLIP
DED1
DED2
Pocket
FL motif
p18
p12
DED1
DED2
1
2
3
4
B
F122G/L123G
1
2
3
4
No caspase-8 chain formation
No caspase-8 recruitment
Reduced caspase-8 recruitment, and no caspase-8 chain formation

Figure S4 Putative models for the recruitment of procaspase-8 and other DED-only proteins to the DISC.

(A) Upon stimulation by the appropriate death ligand, death receptors recruit FADD via their death domains (DDs). FADD in turn recruits procaspase-8 (or procaspase-10 or c-FLIP) through a death effector domain (DED)-mediated interaction. FADD may initially recruit one molecule of procaspase-8 via a single interaction with either DED1 (model 1; see also Figure 7A) or DED2 (model 2). Alternatively, FADD may recruit two DED-only proteins, one via DED1 and the other via DED2 (model 3). In a final variation, FADD from different receptor complexes may self-associate and then caspase-8 chain formation could proceed from each FADD molecule (model 4). We propose that, once bound to the DISC, DED-only proteins are then able to use their exposed binding sites (pocket or FL motif) to recruit additional molecules to produce a chain enabling dimerization and full activation of caspase-8/10. (B) Mutation of the FL motif in caspase-8 DED2 (F122G/L123G) will affect each model differently. In model 1, DED2 mutant caspase-8 is still recruited to FADD via DED1 but is unable to form a chain, whilst in model 2, DED2 mutant caspase-8 cannot be recruited to FADD. In models 3 or 4, DED2 caspase-8 recruitment to FADD is reduced, as only DED1 can interact with FADD, and caspase-8 chains cannot form through mutated DED2.
